# Experimental Investigation of Polymer-Coated Silica Nanoparticles for EOR under Harsh Reservoir Conditions of High Temperature and Salinity

**DOI:** 10.3390/nano11030765

**Published:** 2021-03-18

**Authors:** Alberto Bila, Ole Torsæter

**Affiliations:** 1 Department of Chemical Engineering, Faculty of Engineering, Eduardo Mondlane University (EMU), Av. Moç. km 1.5, Maputo CP. 257, Mozambique; 2 Centre of Studies in Oil and Gas Engineering and Technology, Eduardo Mondlane University (EMU), Av. Moç. km 1.5, Maputo CP. 257, Mozambique; 3 PoreLab Research Centre, Department of Geoscience and Petroleum, Norwegian University of Science and Technology (NTNU), S. P. Andersens veg 15a, 7031 Trondheim, Norway; ole.torsater@ntnu.no

**Keywords:** polymer-coated nanoparticles, core flood, EOR, interfacial tension, wettability alteration, nanoparticle-stabilized emulsion and flow diversion

## Abstract

Laboratory experiments have shown higher oil recovery with nanoparticle (NPs) flooding. Accordingly, many studies have investigated the nanoparticle-aided sweep efficiency of the injection fluid. The change in wettability and the reduction of the interfacial tension (IFT) are the two most proposed enhanced oil recovery (EOR) mechanisms of nanoparticles. Nevertheless, gaps still exist in terms of understanding the interactions induced by NPs that pave way for the mobilization of oil. This work investigated four types of polymer-coated silica NPs for oil recovery under harsh reservoir conditions of high temperature (60 ${}^{\circ }$C) and salinity (38,380 ppm). Flooding experiments were conducted on neutral-wet core plugs in tertiary recovery mode. Nanoparticles were diluted to 0.1 wt.% concentration with seawater. The nano-aided sweep efficiency was studied via IFT and imbibition tests, and by examining the displacement pressure behavior. Flooding tests indicated incremental oil recovery between 1.51 and 6.13% of the original oil in place (OOIP). The oil sweep efficiency was affected by the reduction in core’s permeability induced by the aggregation/agglomeration of NPs in the pores. Different types of mechanisms, such as reduction in IFT, generation of in-situ emulsion, microscopic flow diversion and alteration of wettability, together, can explain the nano-EOR effect. However, it was found that the change in the rock wettability to more water-wet condition seemed to govern the sweeping efficiency. These experimental results are valuable addition to the data bank on the application of novel NPs injection in porous media and aid to understand the EOR mechanisms associated with the application of polymer-coated silica nanoparticles.

## 1. Introduction

Water flooding is the most widely used secondary fluid injection process into an oil-bearing formation, after primary depletion, to improve oil recovery potential. Water is pumped from injection wells, sweeping the oil in the reservoir pores, to the production wells. In this course, water preferentially channels and flows through the high-permeability zones, leaving behind a significant amount of displaceable oil in low permeability-bypassed zones of the reservoir. The reservoir conformance problems manifest themselves or arise due to the contrasts in reservoir fluid properties, heterogeneity of reservoir permeability, fluid mobility contrast, etc. [1]. Moreover, during the productive life of an oilfield, these problems cause the oil to be easily trapped by capillary forces and/or bypassed by the oil recovery-drive fluid, resulting in excessive production of water and, therefore, resulting in poor sweep efficiency. The conformance problems, coupled with the scarcity of new oil field discoveries are the most pressing reasons for the emergence of new oil recovery technologies, aiming to (i) extract about 50% of the original oil in place (OOIP) that is left in the reservoir after primary and secondary recovery stages [2], (ii) increase oil production rates from existing fields, and (iii) fill the gap between energy supply and demand worldwide.

**Figure 1:**
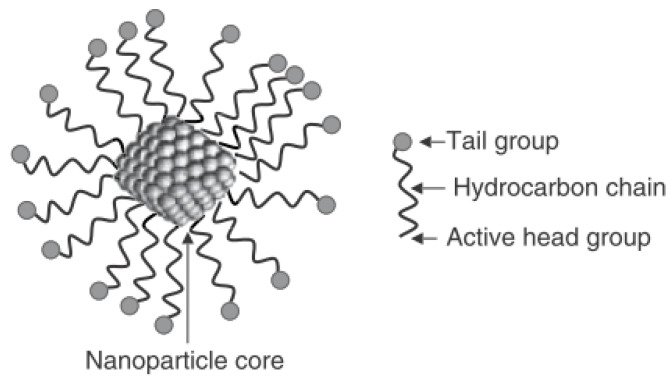
Schematic of a nanoparticle, showing the core and the shell [4].

Nanotechnology has shown great potential to solve some of the above problems and increase profitability for the oil and gas companies. The building block of nanotechnology is the nanoparticle and it operates at the nanoscale. Nanoparticles (NPs) are defined as a collection of atoms bonded together with diameter size in the range of 1 to 100 nm [3]. Figure 1 illustrate a nanoparticle, it is composed of a *core*, the inner material, and the *shell*, the outer layer. The *core* determines the properties of a NP, whereas the *shell* provides a protective membrane and determines the solubility or binding affinity of the NPs with other materials [4]. For oil recovery applications, NP is designed to be wetted by both phases, thus be partly hydrophilic and partly hydrophobic [5]; together with the small size and the large surface area, NPs can have a profound displacement effect on the oil recovery-drive fluid.

The small particle size confers excellent mobility properties; hence, NPs can propagate deeply in the reservoir and increase oil recovery from thief zones and/or from bypassed zones with little retention. With small size, NPs have a large surface area and thus, a large contact area in the swept areas [6] and improved chemical reactivity properties. Therefore, NPs are suitable candidates for changing the reservoir rock and fluid properties and aid in the sweep efficiency of the injected fluid. In order to be successful for EOR applications, nanoparticles must (i) be stable in high salinity, high temperature, and high pressure reservoirs, (ii) propagate long distance between the injection and production wells with little retention, (iii) adsorb on the desired critical sites of the reservoir, such as the oil/water and fluid/rock interfaces, and (iv) prevent over-deposition on the pores [7].

One approach to achieving the above conditions and tailor the properties of NPs to improve their sweep efficiency, especially in harsh reservoir environments, is to covalently attach polymer molecules to the surface of the nanoparticles. The resulting novel polymer-coated NPs have received a wide interest in the oil and gas industry due to their improved solubility and stability, greater stabilization of emulsions and improved mobility through porous media [8,9]. Few studies have reported such characteristics for oil recovery. Rodriguez et al. [10] and Zhang et al. [11] reported that SiO${}_{2}$ NPs coated with polymer molecules have a remarkable transport behaviour through reservoir pores of various permeability with little retention due to their reversible adsorption on the rock surface. Ponnapati et al. [12] experimentally found that polymer (*Poly-(oligo(ethylen oxide) monomethyl ether methacrylate)*-modified SiO${}_{2}$ NPs could mobilise residual oil and yield 7.9% of the OOIP. Behzadi and Mohammadi [13] argues that polymer-coated silica NPs can modulate oil/water interfacial tension and change the wettability of an oil-wet glass micromodel to a more water-wet state, which can confer a greater EOR effect than unmodified silica nanoparticles. Choi et al. [14] reported that grafting polymer shell layers on the surface of silica NP can improve stability in harsh reservoir conditions. Their core flooding tests could achieve 74.1% of the OOIP with the modified NPs, which was quite comparable to plain water flood (68.9%) and unmodified silica NP. The authors associated the EOR effect to the NPs’ ability to decrease the injection pressure; the authors argued that displacement pressure decrease is related to the formation of a wedge film between the oil and the rock surface. More recently, Bila et al. [15,16], Bila and Torsæter [17] carried out a series of flooding experiments with polymer-coated SiO${}_{2}$ NPs in Berea sandstone core plugs. Their studies revealed an incremental recovery ranging from 2.6% to 14% of the OOIP. The authors found that the displacement efficiency of polymer-coated SiO${}_{2}$ NPs is better in water-wet cores than that achieved with induced neutral-wet core plugs.

In summary, the application of NPs, at laboratory scale, have shown an incremental recovery of oil ranging from 5 to 15% of the OOIP [3], the highest reach is 32% of the OOIP [18,19]. The most frequent range has been 5% of the OOIP [20]. Obviously, oil recovery by NPs is a complex phenomenon, partly because the reservoirs are unique and have different characteristics. On the other hand, the variability of experimental approaches in assessing the efficiency of nanoparticle recovery ends up with variable results and variable interpretation of the causes of oil displacement.

Following the encouraging results, studies have demonstrated marvellous efforts to understand the EOR mechanisms of nanofluid flooding. The change in reservoir wettability and the reduction of the interfacial tension are the two well accepted mechanisms of NPs [20,21]. Nanoparticles can alter the reservoir’s wettability by (i) adsorbing on the reservoir rock to develop a new surface roughness [22,23], destabilizing oil films and desorbing it from the surface, and (ii) applying the structural disjoining pressure mechanism [24]. The adsorption of charged NPs can change the reservoir’s wetting properties by forming hydrogen bonds with water molecules, there on attracting water molecules to the surface while lifting oil from it [18,25]. Further, the adsorption of NPs can reduce the interfacial energy between the rock surface and the oil, which disrupts whatever molecular attachment amongst the rock surface and the oil molecules attached to the surface [25]. Nanoparticles can also adsorb onto oil/water interface and decrease the interfacial tension between the two phases. For this, the NPs form a mono-layer that replaces the existing oil/water interface, acting as a mechanical barrier and bring the two phases together [26]. Moreover, depending on the hydrophobicity nature of NPs, they can irreversibly adsorb to the oil/water interface. There, the formed denser layer of NPs can protect oil droplets from flocculation and coalescence via steric mechanism to result in the stabilization of emulsion droplets by nanoparticles [9,27]. These emulsions (Pickering emulsions) can travel through the pores of the reservoirs with minimal retention and increase oil recovery [27].

Aside from the above mentioned mechanisms, NPs can block reservoir pore-throats larger than their size and increase oil recovery via log jamming mechanism [28,29]. Nanoparticles can increase the viscosity of EOR fluid and reduce the viscosity of the heavy oil, which can be reflected in a favourable mobility of the displacing and displaced phases, respectively, for oil recovery.

This work aims to expand our previous works [15,16] and attempts to investigate the recovery mechanisms of polymer-coated silica NPs under harsh reservoir conditions of high temperature and salinity. The goal is to provide additional experimental results to the data bank on the nanoparticles for enhanced oil recovery projects, while paving the way to improve our understanding of the underlying EOR mechanisms associated with silica nanoparticles.

## 2. Experimental Materials and Methods

### 2.1. Nanoparticles and “Simulated” Seawater

**Figure 2:**
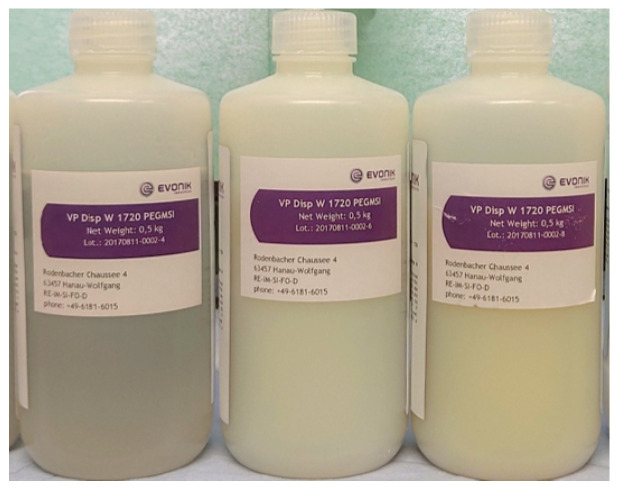
Concentrated aqueous solutions of silica nanoparticles.

Nanostructured products from Evonik Industries AG were used for oil recovery experiments in this work. The nanostructured materials were supplied as special research and development (R&D) laboratory products, hereafter simply referred to as nanoparticles. They were supplied to us in small containers as AERODISP^®^, which is AEROSIL^®^ particles in liquid solution as shown in Figure 2. They are spherical and amorphous products mainly composed of silicon dioxide (SiO${}_{2}$ > 98.3%); additional components are aluminium oxide (Al${}_{2}$O${}_{3}$) and mixed oxides (MOX). The surface of NPs were coated with Poly (methacrylate) based molecules to improve dispersion stability and lower the hydrophilicity nature that characterizes typical silica materials to form polymer-coated/functionalized nanoparticles. Properties of the particles are given in Table 1. The size of the nanoparticles was measured using the dynamic light scattering technique.

The simulated seawater was prepared with ions typical of sea water (Na${}^{+}$, Ca${}^{2+}$, Mg${}^{2+}$, SO${}_{4}$${}^{2+}$, K${}^{+}$, etc.), by mixing salts with distilled water, following the homogenization process. The total of dissolved salts was 38,318 ppm. For oil recovery experiments, the concentrated solutions of nanoparticles were diluted to 0.1 wt.% concentration with simulated seawater. The resulting nanofluid solutions were stirred using a magnetic stirrer for at least 30 min to prevent aggregation/agglomeration of particles. The properties of water and nanofluids are given in Table 2, and were measured in our previous work [16]. The density and viscosity were measured at 60 ${}^{\circ }$C with an Anton Paar density meter DMA^TM^ 4100 M series and Anton Paar Rheometer, respectively. The pH of the solutions was measured by a pH Meter (model pH 1000 L, phenomenal^®^).

### 2.2. Non-Wetting Phase

Crude oil obtained from a field in the North Sea was used as the non-wetting phase, it had a viscosity of 6 cP and 33 °API gravity at at 60 ${}^{\circ }$C. The crude oil SARA (Saturates, Aromatics, Resins, Asphaltenes) analysis is provided in Table 3. It is classified as a light oil with asphaltenes content of 0.18 wt.%. It was filtered twice through a 5 μm Millipore to remove suspended particles and preserve original composition. The density and viscosity were measured with the same instruments used in Section 2.1. Normal decane with density and viscosity of 0.73 g/cm${}^{3}$ and 0.92 cP at 20 ${}^{\circ }$C, respectively, was used in the wettability experiments.

### 2.3. Preparation of Porous Medium

Eight core plugs were drilled from Berea sandstone block and prepared to have similar diameter (3.75 cm) and length (10 cm). The bulk mineral composition was measured using X-ray diffraction (XRD) on five core plugs. All samples were composed of 93.7 vol % quartz, 5 vol % of Microcline (Alkali feldspar) and 1.3 vol % Diopside. They are classified as homogeneous and strongly water-wet rocks. The cores were cleaned with methanol using Soxhlet extractor and dried at 60 ${}^{\circ }$C for ≈3 days. Porosity was measured by imbibition method and permeability by Darcy’s law using a constant head permeameter, flowing nitrogen gas through the core. The gas permeability obtained was then corrected for the Klinkenberg effect to reflect the liquid’s permeability. The core properties are given in Table 4.

The cleaned and dried core plugs were saturated with reservoir simulated formation water using a vacuum pump with a chamber pressure set at 100 mbar for three hours. Then, the core plugs were soaked in the same water for at least 10 days to attain ionic equilibrium with the rock constituents.

Primary drainage was conducted by injecting crude oil into the cores to displace the water and set initial water saturation (S${}_{wi}$). Next, the cores with S${}_{wi}$ were submerged in metallic containers filled with the same crude oil used to displace the water and soaked for seven months at 80 ${}^{\circ }$C. The aim was to decrease the water wetness in the cores. The aging time and the high temperature can break the water films on the surface, facilitating the adsorption of polar components of crude oil to change the rock wettability. The results of the aging process are presented in Section 3.2.4 and show that wettability of the cores has been changed from a strongly water-wet surface to a neutral-wet state.

### 2.4. Displacement Tests

**Figure 3:**
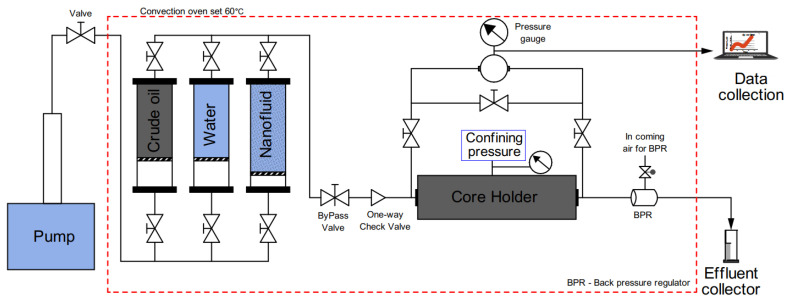
Schematic diagram of core flooding experiment.

Oil displacement tests were carried out at the reservoir temperature (60 ${}^{\circ }$C) in aged core plugs to determine the EOR potential of silica based nanofluids. A schematic of the core flooding apparatus is shown in Figure 3. It is comprised of an injection pump, three tanks each containing crude oil, water and nanofluid. The reservoir tanks were assembled vertically inside an oven set at 60 ${}^{\circ }$C. A check valve and back pressure regulator (set to 5 bars) were used to prevent any back flow of produced fluids and keep the system pressure constant during the experiments. The core was loaded in the Hassler core-holder and oriented horizontally under confining pressure held within 18–22 bars. The crude oil was injected at low rate (0.02 mL/min) to eliminate any air bubbles, until the temperature in the reservoir stabilized at 60 ${}^{\circ }$C. To simulate oil production of a reservoir: (i) conventional water-flood was conducted at a constant reservoir flow rate, 0.2 mL/min, until there was no oil production for 1-pore volumes (PVs). Next, the flowrate was increased ten-fold (bump flood) for 1-PV in order to mitigate capillary end-effects, establish water flood residual oil saturation and ensure that any production would result from the injection of nanofluid. (ii) the injection was continued with nanofluids at the constant flow rate until there was no more oil production at core outlet for ≈3-PVs; likewise, the bump flood was applied for 1-PV. To study the behaviour of the injection fluid flow through the pores, two pressure sensors were connected at the entrance and exit of the core-holder; the differential pressure of the fluid across the core was recorded using a computer.

### 2.5. Interfacial Tension Measurement

Interfacial tension (IFT) is the most important property that characterises the interface of two immiscible or partially miscible fluids that are in contact. Accordingly, it influences the extraction of crude oil from reservoir pore spaces. Interfacial tension can be measured by a variety of methods. A spinning drop method, SVT20N (Data Physics) video tensiometer, was used in this work. The crude oil was injected drop-wise into a capillary tube filled with water or nanofluid. Then, the apparatus was set a 60 ${}^{\circ }$C and the tube rotated at a speed held within 6000–8000 rpm until the equilibrium was reached; then, the static IFT value was read off. The average refractive index of the continuous phases was 1.338.

### 2.6. Spontaneous Imbibition Tests

The spontaneous imbibition (SI) tests provide a qualitative measure of the ability of the wetting-phase to displace the non-wetting phase under static conditions [30]. Generally, the SI tests are performed in visual glass with graduated tube on top called Amott cell. An oil-saturated core plug (at S${}_{wi}$) is place in the cell filled with water. Free-oil displacement by water is expected to take place over a period of time. The produced oil is collected on the top of graduated tube until equilibrium is reached. The dynamic imbibition is established as cumulative oil production versus time to interpret the change in wettability. In this work, the Amott water index (I${}_{w}$) was also calculated to validate SI tests. At equilibrium, the volume of oil produced by SI is V${}_{o1}$. The remaining oil in the core was forcibly displaced by injecting water; the produced oil is V${}_{o2}$. The Amott water index is calculated as: I${}_{w}$ = V${}_{o_{1}}$/(V${}_{o_{1}}+V_{o_{2}}$). The closer to 1.0 the I${}_{w}$ is, the strongly water-wet the rock system is; smaller values of I${}_{w}$ indicate less water-wetting preference. It is worthy to mention that the core plugs were injected with decane before and after the nanofluid-EOR tests to set S${}_{wi}$; then the imbibition tests were followed. This procedure aimed to study the wetting conditions that can be achieved after the interaction between the NPs and crude oil/water/rock system. The results are used to interpret oil displacement mechanisms of polymer-coated silica NPs.

## 3. Results and Discussion

### 3.1. Nanoparticle’s Oil Recovery

Most oil reservoirs are characterised by high pressure, high temperature, high salinity and an uneven properties [8,31,32]. Thus, EOR technologies must be designed to operate nearly under identical conditions to provide realistic results. Presently, an attempt was made in this direction, carrying out the EOR experiments at the reservoir temperature of 60 ${}^{\circ }$C, and dispersing the nanofluids in typical reservoir brine. The cores aged to less water-wet state. A bump flood was applied at the end of the low rate flood to ensure that any additional production would be a result of the nanofluid injection.

**Figure 4:**
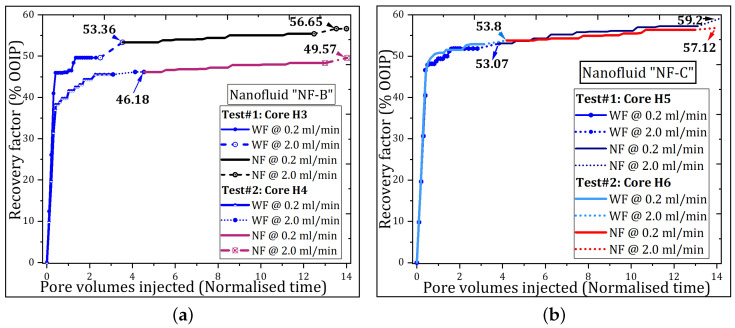
Effect of nanofluid injection on oil recovery during normal low rate and bump rate injections: (**a**) Sample NF-B: Test#1 the first oil production occurred ≈1.8 PVs and the RF ≈ 0.5% OOIP; Test#2 produced ≈ 0.5% OOIP at 1 PV. (**b**) Sample NF-C: Test #1 the oil production occurred at ≈2.2 PVs and the RF ≈ 2.3% OOIP; Test #2 the first production occurred at ≈1.3 PV and the RF ≈ 0.3% OOIP.

The oil recovery performance for the selected nanofluids is shown in Figure 4. Note that each nanofluid sample was tested twice. Figure 4a,b show the oil displacement profiles for the nanofluids with the smallest particle size (32 nm) and the largest particle size (218 nm), respectively. The oil recovery from the water flood (WF) is given by the blue curves, sequentially for (i) low rate (continued lines), and (ii) tenfold increase in flow rate for 1 PV (dashed lines). Then, the nanofluid was injected targeting water flood residual oil saturation. Likewise, the continued and dashed lines indicate the recovery of oil during the low rate and the high rate, respectively. The water flood conducted over the aged core samples exhibited an early water breakthrough, but accompanied with prolonged periods of co-production of water and oil. This displacement behavior conferred a greater oil recovery at the end of water flood, which is an agreement with previous studies [33,34]. The recovered oil, during the bump flood stage, seemed insignificant, except for the core H3 with 3.73% of the OOIP. This oil production can be attributed to the capillary end-effects and/or capillary stability. When capillary stability is not guaranteed during the low-rate displacement stage, the oil can snap off and easily become trapped in the larger pores as water saturation increases in the pores. These disconnected oil drops can be easily produced by increasing the flowrate. Experimental tests suggest that the capillary end-effects are less pronounced in intermediate rock systems [15,35], such as those used in this work.

It is important to note that the current results indicate that on a laboratory scale, any EOR fluid must be performed after many pore volumes of water have been injected in the cores at low rate to achieve capillary stability and establish an adequate residual oil saturation. With this procedure, if the EOR fluid is successful in the laboratory, it is more likely to be successful in the field as well.

**Figure 5:**
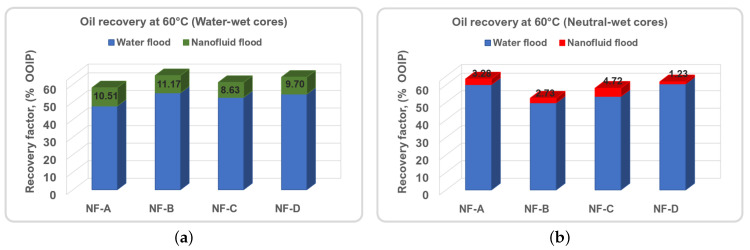
Comparison of average oil recoveries from various nanofluid floods: (**a**) Water-wet cores [16], and (**b**) Neutral-wet Berea core (present study).

Nanofluid was injected to interact with the crude oil/rock/water system. The occurrence of oil at the exit of the core during the nanofluid injection stage was observed after 1 or 2 PVs (see Figure 4). This shows the time dependence of such interactions between the NPs and the crude oil/water/rock system towards improving the microscopic sweep efficiency of the oil-drive fluid. The interactions can be of particle adsorption type on the crude oil/water/rock interfaces. There on, the NPs can decrease the interfacial energy and change the wetting properties of the rock surface, and increase oil recovery. All results are summarised in Table 5. The average recoveries are shown in Figure 5 for comparison with the previous study. Total water flood oil recoveries varied in between 46.18% and 66.75% of the OOIP. This variation can be due to saturation profiles, pore structures, viscosity ratio and experimental errors. On average, 10-PVs of nanofluids were injected. This resulted in an incremental recovery ranging from 1.51 to 6.13% of the OOIP. The twin core plugs produced comparable recovery factors and the differences are in the margin of experimental error. The displacement efficiency (E${}_{D}$) evaluated by Equation (1) shows the effectiveness of polymer-coated silica nanofluids in mobilising water flood residual oil at pore scale under harsh reservoir conditions.

(1) $$E_{D}=\left[1-\left(\frac{S_{or_{2}}}{S_{or_{1}}}\right)\right]\times 100\%$$

Here, the S${}_{or_{1}}$ and S${}_{or_{2}}$ is the residual oil saturation after water- and nanofluid-flooding, respectively.

The temperature seemed to have minor influence on oil recovery in neutral-wet cores, unlike in water-wet cores. Figure 5 illustrates the average oil recoveries for comparison purposes obtained in water-wet and neutral-wet Berea cores conducted under similar procedure and conditions. Figure 5a shows the nanofluid flooding results in water-wet systems and were extracted from Bila et al. [16], while the current results are given by Figure 5b and were calculated from Table 5. Clearly, the results indicate a superior oil recovery behaviour in water-wet Berea sandstone with the injection polymer-coated silica NPs. This may be associated with entrapping phenomenon in both rock systems during water flooding stage. In water-wet rocks, water fills the smaller pores and retains a considerable amount of the oil in the larger pores after water flooding which is amenable to EOR process. In contrast, neutral-wet cores tend to recover considerable amount of oil, despite the early water breakthrough. In addition, oil is trapped in the smallest pores and the residual oil saturation would resist mobilisation.

**Figure 6:**
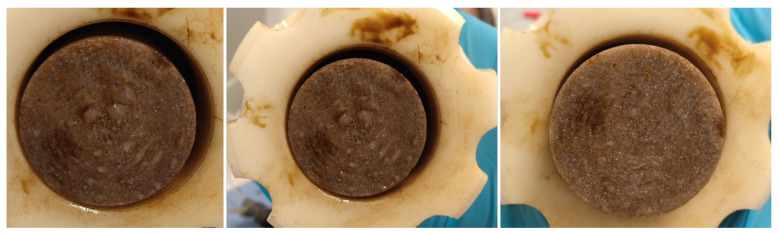
Physical filtration and formation of nanoparticle “cake” at core inlet during nanofluid flooding.

Aside from the temperature, the size of the nanoparticles seemed to influence the displacement efficiency; largely due to stability issues. Particularly, samples NF-C and NF-D with the largest particle size aggregated at core entrance during the EOR experiments. This resulted in physical filtration and the formation of nanoparticle “cake”, shown in Figure 6. This was also observed in our previous work [16] but with water-wet Berea core plugs. However, the resultant layer of NPs appeared noticeably thicker in water-wet rocks, resulting in higher displacement pressures than in neutral-wet rock systems. This may indicate that polymer-coated NPs are more effective in improving the fluidity of the particles through neutral-wet Berea pores than in water-wet Berea pores.

### 3.2. Mechanisms behind EOR by Nanoparticles

#### 3.2.1. Effect of Nanoparticles on Viscosity of Injection Water

Together with interfacial tension, the viscosity provides a qualitative measure of how, and to what extent, oil will flow through porous media [36]. The research have reported tremendous increase in the viscosity of the injection fluid in the presence of NPs, which can significantly improve oil sweep efficiency. The size and concentration of the NPs are the main parameters that affect the viscosity of the injection water [8,25]. An increase in the viscosity of injection fluid is reflected in the mobility of adjacent fluid molecules around the nanoparticles. For instance, highly concentrated nanofluid can give a uniform displacement front [12,25], which is favourable for increasing oil sweep efficiency.

In the present work, nanoparticles at 0.1 wt.% had negligible effect on the viscosity of injection seawater. The viscosity of the nanofluids varied from 0.51 to 0.67 cP compared to 0.53 cP of reference injection water. Hence, neither the concentration of polymers, as coating materials, nor the concentration of NPs was enough to generate a significant viscosity change in aqueous solution. The results lead to a similar conclusion to the previous study by Metin et al. [37], where low concentrations (≤5 wt.%) of NPs have been shown not to affect aqueous viscosity. Therefore, we expect oil recovery to be largely influenced by factors other than viscosity.

#### 3.2.2. Reduction in Interfacial Tension (IFT)

Normally, NPs are designed to be wetted by both phases, thus being partly hydrophilic and partly hydrophobic, to allow for a better reduction in IFT [5] and decrease capillary forces. At low capillary forces, the dynamic displacement lengthens the oil droplets within the pores; then the oil drops break into smaller ones to flow within the waterbed to the production wells.

**Figure 7:**
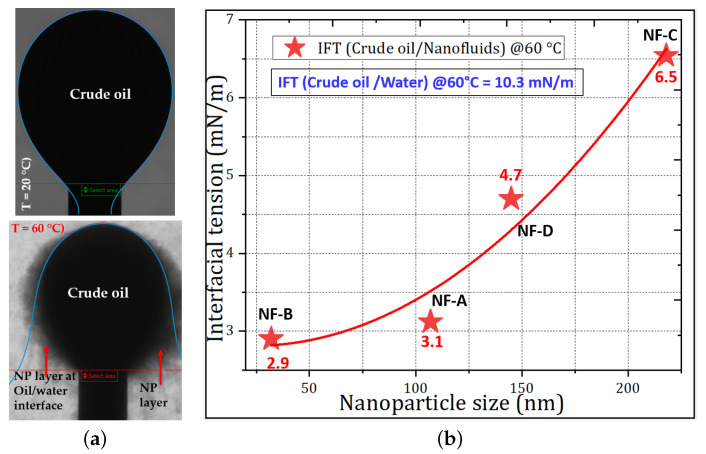
Dynamic measurement of the IFT between crude oil and nanofluids (0.1 wt.%): (**a**) Pendant drop method: Crude oil drop on top of a J-shape syringe needle at 20 ${}^{\circ }$C (top) and at 60 ${}^{\circ }$C (bottom). At 60 ${}^{\circ }$C, the nanoparticles self-assembled onto the oil/water interface [16]. (**b**) Variation of the IFT measured with Spinning drop technique (at 60 ${}^{\circ }$C) with nanoparticle size.

Bila et al. [16] found that IFT measurement with the Pendant drop method would not provide reliable results because, at high temperature, the polymer-coated SiO${}_{2}$ NPs were attracted to the oil/water interface (see Figure 7a). From then on, making nanofluid solutions opaque before settlement due to gravity forces. Therefore, in this work, the IFT was obtained by analyzing the surface of the oil droplet immersed in a rotating capillary tube using a high-resolution camera and image analysis software. The use of a small volume of fluids in the capillary tube and the speed of rotation probably hindered the formation of large NP aggregates, allowing the measurement of IFT. At the equilibrium, IFT values obtained by the spinning drop method are given in Figure 7b as a function NP size.

The IFT between crude oil and water, at 60 ${}^{\circ }$C, was 10.3 mN/m [16]; in the presence of NPs, IFT decreased to 6.5 to 2.9 mN/m. Furthermore, Figure 7b shows that the reduction is dependent on the size of the nanoparticles. The smallest NPs tend to exhibit a high affinity capacity to adsorb at the interface, coming into contact with a large surface area; consequently, the NPs had the largest IFT reduction compared to the larger particles. For example, the NF-B sample with the smallest size (32 nm) reduced the IFT by 71.8% compared to the 36.9% reduction by the largest NF-C sample. This can be associated with the increase in repulsive forces between the smallest NPs, resulting in a greater disjoining pressure between the two-phase molecules [38]. In addition, the IFT reduction can be due to the surface-active polymer molecules surrounding the surface of the NPs that provide steric repulsive forces and stabilization of the NPs suspension. Comparing the results of the IFT data from previous studies at room temperature [15,17], for the same NPs, the greatest IFT reduction is achieved at elevated temperature. This indicates that the wetting properties and the binding energy of the NPs to the oil/water interface increase with temperature. This has been attributed to the intensification of Brownian motion, which increases the particle collision and reduces intermolecular interactions between oil/water [26]. However, the displacement of oil at elevated temperature still has a multiple effect on IFT, the formation of aggregates/agglomerates of NPs, the loss of NPs at the interface and the inhibition of NPs from performing their designated surface functions at the interfaces and compete for poor performance of nanoparticles.

According to Sheng [39], significant oil recovery is achievable by reducing IFT to an ultra-low value of 10${}^{-3}$ mN/m. However, the reduction in IFT, even below the critical value, can still play a significant role in EOR; can alter the distribution of oil in the pores [40], can contribute to the generation and stabilization of emulsions by nanoparticles [8].

#### 3.2.3. Formation of Emulsions Stabilized by Nanoparticles

**Figure 8:**
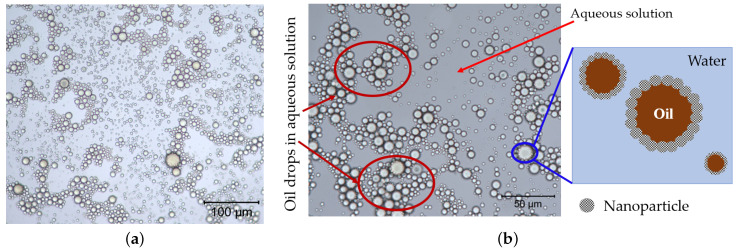
Visualisation of oil-in-water emulsion droplets from core flood effluent: (**a**) 20× magnification image, and (**b**) 50× magnification image.

A reduction in interfacial tension can create favorable conditions for the generation and stabilization of emulsion during the process of oil recovery by nanofluids. This premise was studied by increasing injection flow rate at the end of low rate injection. High flow rate can provide an extra energy necessary to break up oil phase and allow for NPs to adsorb to the fluids interface [41], thus generating emulsions. The produced core flooding effluent was then visualised using a high-resolution microscopic camera; the magnified images of the flooding effluent are presented in Figure 8. The images illustrate oil drops dispersed in aqueous solution in the presence of nanoparticles, indicating that residual oil was produced as oil-in-water emulsions.

In Figure 8, the adsorption of silica NPs seemed to create a rigid layer on the surface of the oil droplet, temporarily stabilising oil droplets against flocculation and coalescence via steric mechanism in the aqueous phase. The size distribution of the generated emulsions was not determined to compare with pore-throat sizes; however, Arab et al. [42] explain that oil drops created and stabilized by tiny NPs are small and possess a considerably lower viscosity than oil drops. Herewith, nanoparticle-stabilized emulsions, owing to their small size, can travel more easily through tiny reservoir rock pores with minimal retention, improved kinetic stability and negligible gravitational separation, resulting in increased oil recovery [8,23,26,43]. This profile mechanism (stabilisation of emulsions) probably occurred thanks to the polymer shells, as they temporarily prevented the coalescence of the oil drops. The phase separation of water and oil occurred approximately half an hour in the separator flask; the separated volumes were measured after two days.

The images showed in Figure 8 refers to emulsions generated in-situ by samples NF-A and NF-B; in contrast, samples NF-C and NF-D with the lager particle size generated weak emulsions. Among other factors, this shows that nanoparticles in the aggregate state are weak emulsifying agents. This has been attributed to either the kinetics of particles adsorption to the oil/water interface, being reduced by the presence of aggregated particles or networks, thus hindering the formation of emulsions [44].

#### 3.2.4. Change in Rock Wettability and Surface Roughness

**Figure 9:**
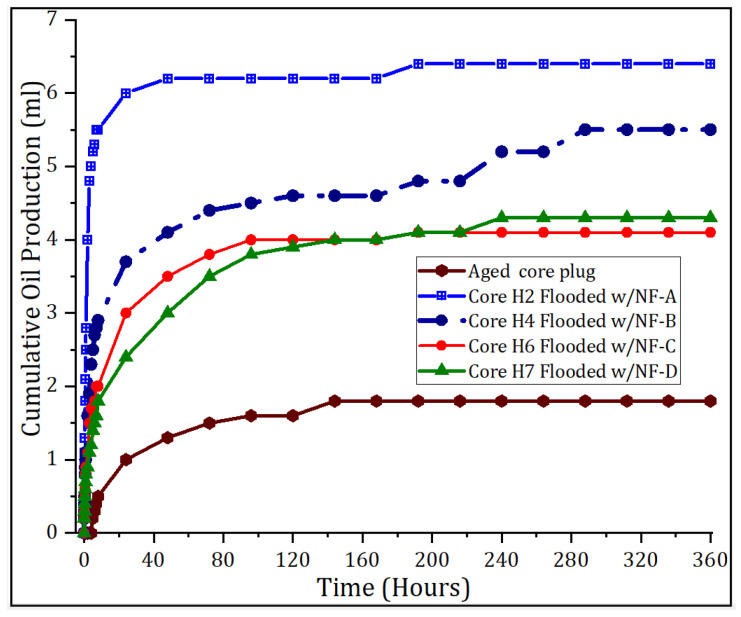
Spontaneous imbibition curves, before and after nanofluid core flooding.

Nanoparticles target improving oil recovery by changing the surface roughness of the reservoir pores and thus altering its wettability to more water-wet state. The degree of water-wetness that can be achieved largely depend on how the NPs affect the crude oil/brine/rock properties [30]. Presently, we conducted spontaneous imbibition (SI) tests to evaluate such interactions induced by nanoparticles after nanofluid oil recovery experiments. In SI tests, the water will imbibe in the reservoir pores and displace the oil, if the reservoir is likely water-wet, as it has a positive capillary pressure [30]. The results of SI tests are presented in Figure 9. Prior to nano-EOR tests, the cores were aged 7 months in crude oil; post aged core wettability was evaluated by SI using one core plug. The lower curve, in Figure 9, shows the water imbibition performance in the aged core; there was no appreciable oil production before 10 days. Its Amott wettability index was −0.08, indicating neutral-wet condition. Additional cores aged under similar conditions were used for recovery tests, assuming that they retained the same neutral-wettability. After nanofluid core flood ceased production, the wettability alteration due to the adsorption of NPs was investigated. Figure 9 shows the cumulative oil production by water imbibition as a function of time. Depending on the particle size and particle retention in the pores, rock pore size distribution, etc., varied water imbibition profiles were achieved, but all showed an improved rate of water intake. That is, the water spontaneously invaded the rock pores and displaced the oil significantly, shortly after the placement of the core in the Amott cell. In contrast to the reference core where the oil production by water imbibition occurred from the tenth day, and reached stability 5 days later; thereafter, no oil production was observed. Part of the initial oil production of the aged core was the oil strongly attached to its surface after aging process. The jump in oil recovery, at 120 h, is because the cell was slightly shaken. In Figure 9, cores H2 and H4 were treated/injected with samples NF-A and NF-B, respectively. Its imbibition curves indicate superior oil recovery behavior by SI compared to other; this predicts the greater ability of the NPs to change oil-wet pores to water-wet state and increase oil recovery in a timely manner. It is worthy to mention that NF-A and NF-B had the smallest NP size and were stable throughout the duration of nanofluid flooding experiments. Therefore, with small size, NPs could contact most of the pore spaces to change its wettability.

In Figure 9, we see that greatest recovery of oil occurred within 40 h; after that, a later, an increase in oil recovery was observed. This indicated that the wettability was changed with time, in some parts of the pores, due to the nanoparticles. At equilibrium, the Amott water index (I${}_{w}$) was determined and varied from 0.67 to 0.77. The rate of SI and the I${}_{w}$, both indicate an alteration in core wettability to increasing water-wet condition. The capillary pressure that was negative in oil-wet pores was changed to positive values due to adsorption of NPs, which led to a stronger water imbibition in smallest pores. These results are in line with our previous study [15], and validate the ability of polymer-coated silica nanoparticles to alter the wettability of the rock to favor EOR process.

The mechanism behind the wettability alteration in oil-wet surfaces is still complex. However, it is probably associated with the adsorption of silica NPs to the crude oil/water/rock interfaces due to the attractive forces originating from dipole-dipole interactions [18,25] and structural disjoining pressure. These interactions can develop new surface roughness [23], decrease interfacial energy between the rock surface and the water [25], and destabilise oil films attached on the surface. Consequently, water-wet surfaces are created in the pores and greater capillary pressure ensures greater efficiency in oil recovery by nanoparticles.

#### 3.2.5. Nanofluid Displacement Pressure

To better understand the mechanisms of oil recovery by nanofluids, dynamic curves of the pressure difference through the core plugs were recorded and analysed. The premise is that nanofluids can change the flow pattern or the pore volume available for flow; for this, the resulting differential pressure must be greater than the water flood pressure. Under these conditions, mobilization of residual oil can occur through so-called log-jamming effect.

**Figure 10:**
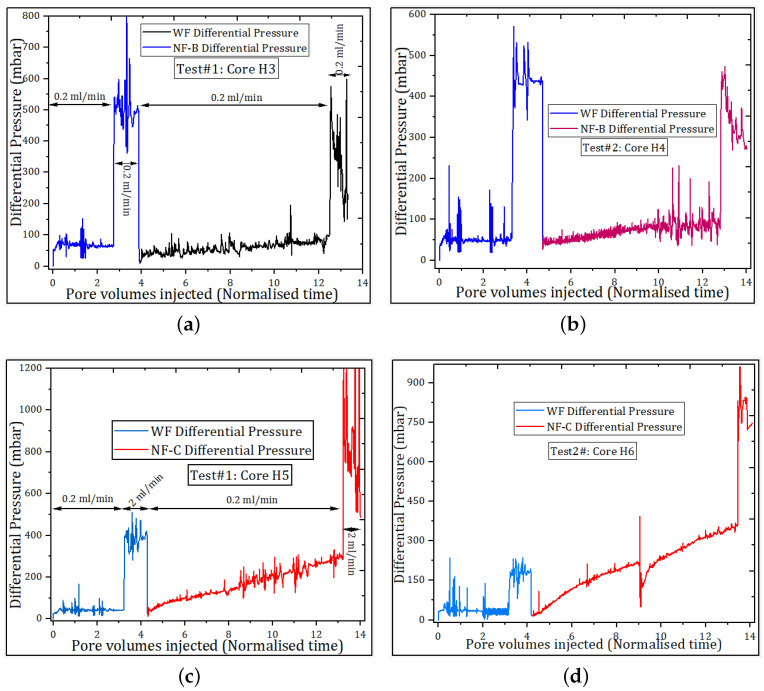
Pressure profiles for water flood (WF) and nanofluid flood oil recovery. (**a**) Test #1: Nanofluid “NF-B” injection showing little effect on water flood pressure; (**b**) In repeated Test #2, the pressure shows a slight increase compared to WF pressure; (**c**) Test #1, the pressure gradually increases with nanofluid “NF-C” and is higher than WF pressure; and (**d**) Similar behavior is observed in Test #2.

Figure 10 shows the change in the flow pattern of the displacement pressure due to nanoparticles. While Figure 10a,b exhibit a slight change in water flood pressure, Figure 10c,d show a notable influence of NPs on the displacement pressure. The pair of the cores flooded with NF-B (Figure 10a,b) exhibited an injection pressure pattern similar to that of cores injected with the NF-A sample; the small size and dispersion stability of both samples (NF-A and NF-B) can be the reasons behind the negligible effect on the water flood displacement pressure. Furthermore, it suggests that both NF-A and NF-B samples can propagate through the pores and assist in oil recovery even in the smallest pores. The transport properties of nanoparticles were probably improved by the surface coating materials (polymer molecules), as they can eliminate electrostatic interactions between NPs and oil-wet surfaces [10,11]. The observed pressure profile mechanism during NP injection is consistent with the generation of in-situ emulsions. The propagation of NPs in smaller pores would release the oil from the surface to flow into the water bed and improve the relative permeability to the oil.

In contrast to samples NF-A and NF-B injection, there was a noticeable increase in water injection pressure when nanofluids with the largest particle size (NF-C and NF-D) were injected into the cores. In that case, the pressure gradually increased up to a point; there, it started to fluctuate (see Figure 10c,d). This displacement pressure pattern is a direct evidence of pore channel plugging. The NPs were partially transported through the pores, while others were retained, blocking the pores and forcing injection water to flow through through the bypassed pores. The diversion of water flow can create additional pressure in the adjacent pores; if the pressure is high enough to generate enough capillary numbers, the bypassed oil can be mobilized to the production wells and increase oil recovery. This displacement mechanism has been reported in the literature [28,29], and in most cases controls the oil displacement in water-wet cores [16,45,46].

Figure 6 shows the formation of NP “cake” as a result of aggregation/agglomeration of samples NF-C and NF-D at core inlet. The “nano-cake” is an evidence that the primary blockage occurred at the entrance of the core, followed by filtration of smaller NPs in response to the applied displacement pressure. Therefore, this phenomenon can be the main reason for the pressure and may not reflect oil recovery by the log-jamming effect.

Inspecting the magnitude of the pressure increase due to NPs in Figure 10, it seems insufficient for the viscous forces to dominate the displacement process over the capillary forces, therefore, in this work, the flow diversion mechanism cannot fully explain the EOR effect due to the injection of nanoparticles.

## 4. Conclusions

This work presents the results of the influence of polymer-coated silica nanoparticles to enhance oil recovery at harsh reservoir conditions of high temperature and high salinity. This included an investigation of the effect of nanoparticle interactions on the crude oil/water/water interfaces, in order to understand the phenomenon of oil recovery. On the basis of the experimental results, the following conclusions can be drawn:

- Polymer-coated silica nanoparticles have shown a strong ability to increase oil recovery after water flooding. The increment recovery was up to 6% of OOIP;
- The nanoparticles can reduce the oil/water interfacial tension at a concentration as low as 0.1 wt.%. The smallest nanoparticle size were more efficient in reducing the tension due to the large contact area;
- The flooding experiments indicated that oil was produced as oil-in-water emulsion droplets; these emulsion droplets were stabilized by the nanoparticles.
- The adsorption of nanoparticles in oil-wet pores can reverse the negative capillary pressure to positive values and change the wettability to water-wet condition;
- The size of nanoparticles and the formation of large aggregates within the pores were observed to increase displacement pressure, resulting in poor oil recovery efficiency;
- Different oil displacement mechanisms, such as reduced IFT, change in wettability, generation of in-situ emulsions and change log-jamming effect can explain the oil recovery phenomenon of polymer-coated silica NPs in intermediate reservoirs. However, the wettability alteration to a more water-wet seemed to govern the oil displacement process.

This paper reveals the potential application of polymer coated silica NPs for oil recovery. Future studies must be directed to improve the stability of the nanoparticles. In addition, studies should also focus on characterizing the charge on the rock surface, the effect of nanoparticles bonding with coating materials (i.e., surface activity and reactivity) to predict their interactions with fluids and rock system during the oil recovery process and probably to determine the contribution of the components involved in the oil recovery process.

## Figures and Tables

**Table 1:** Properties of the aqueous solutions of silica nanoparticles.

Sample	Basis	Modification	Conc., wt.%	Size (nm) ^a^	Size (nm) ^b^
NF-A	SiO${}_{2}$ (sol-gel-cationic)	Polymer	38.6	107	63
NF-B	SiO${}_{2}$ (sol-gel-anionic)	Polymer	26.0	32	38
NF-C	SiO${}_{2}$/Al${}_{2}$O${}_{3}$/MOX	Polymer	21.6	218	155
NF-D	SiO${}_{2}$/Al${}_{2}$O${}_{3}$/MOX	Polymer	25.5	145	135

^a^ Average particle size in concentrated solutions (as received). ^b^ Average particle size measured with NPs diluted to 0.1 wt.% with simulated seawater.

**Table 2:** Fluid properties measured at 60 ${}^{\circ }$C.

Fluid	Density (g/cm${}^{3}$)	Viscosity (cP)	pH
Seawater	1.008	0.53	7.97
Nanofluids	1.007–1.009	0.51–0.67	7.74–8.05

**Table 3:** Properties of the crude oil.

Property	Value	Unit
Saturates	71.57	wt.%
Aromates	20.81	wt.%
Resins	7.44	wt.%
Asphaltenes	0.18	wt.%
Density at 60 ${}^{\circ }$C	0.87	g/cm${}^{3}$
Viscosity at 60 ${}^{\circ }$C	6.0	cP
API gravity	33	deg

**Table 4:** Properties of the rock core plugs.

Core	Porosity, (%)	Permeability, mD	Pore Volume, mL	S${}_{\mathrm{wi}}$ (%)
H1	17.61	332	19.25	24.66
H2	19.55	384	19.06	21.32
H3	17.40	361	19.22	16.24
H4	17.56	434	19.39	16.45
H5	16.70	460	19.83	17.81
H6	17.16	411	19.10	15.00
H7	17.61	425	19.25	14.00
H8	19.42	377	18.78	19.60

**Table 5:** Oil recovery factors (water- and nanofluid-flooding), expressed as % of the OOIP, and residual oil saturation achieved at the end of core flooding in neutral-wet cores.

		Water Flood				Nanofluid Flood					
Core	Fluid	RF1	RF2	RF	S${}_{{\mathrm{or}}_{1}}$	RF1	RF2	RF	S${}_{{\mathrm{or}}_{2}}$	RF${}_{t}$	E${}_{D}$ (%)
H1	NF-A	59.45	2.90	62.35	28.37	2.76	2.07	4.83	24.47	67.18	13.74
H2		57.33	0.67	58.00	33.05	1.73	0.00	1.73	31.68	59.73	4.13
H3	NF-B	49.63	3.73	53.36	39.07	2.05	1.24	3.29	36.32	56.65	7.06
H4		45.56	0.62	46.18	44.97	2.16	1.23	3.39	42.14	49.57	6.31
H5	NF-C	51.84	1.23	53.07	38.57	4.29	1.84	6.13	33.63	59.20	12.81
H6		52.88	0.92	53.80	39.43	2.82	0.50	3.32	36.61	57.12	7.17
H7	NF-D	66.45	0.31	66.75	28.67	1.20	0.30	1.51	27.37	68.25	4.53
H8		53.64	0.80	54.44	37.27	3.05	0.93	3.98	34.08	58.41	8.57

## Data Availability

Restrictions apply to the availability of Nanomaterials used in this study. The nanomaterials were obtained from Evonik Industries as research and development products and may be available on request from the company.
